# Incidence and spectrum of yeast species isolated from the oral cavity of Iranian patients suffering from hematological malignancies

**DOI:** 10.1080/20002297.2019.1601061

**Published:** 2019-04-12

**Authors:** Amir Arastehfar, Farnaz Daneshnia, Shirin Farahyar, Wenjie Fang, Maryam Salimi, Mohammadreza Salehi, Ferry Hagen, Pan Weihua, Maryam Roudbary, Teun Boekhout

**Affiliations:** a Department of Yeasts , Westerdijk Fungal Biodiversity Institute, Utrecht, Netherlands; b Department of Medical Mycology and Parasitology, School of Medicine, Iran University of Medical Sciences, Tehran, Iran; c Microbial Biotechnology Research Center (MBiRC), Iran University of Medical Sciences, Tehran, Iran; d Department of Dermatology, Shanghai Key Laboratory of Molecular Medical Mycology, Shanghai Institute of Medical Mycology, Shanghai Changzheng Hospital, Second Military Medical University, Shanghai, China; e Department of infectious diseases and Tropical Medicine, Faculty of Medicine, Tehran University of Medical Sciences, Tehran, Iran; f Institute for Biodiversity and Ecosystem Dynamics (IBED), University of Amsterdam, Amsterdam, Netherlands

**Keywords:** Oral candidiasis, hematological malignancies, Nystatin, Fluconazole, 21-plex PCR, and MALDI-TOF MS

## Abstract

**Background:** Oral candidiasis (OC) has a profound effect on the life quality of immunocompromised patients, such as those undergoing chemotherapy. **Objective:** Systematic investigation of clinical outcome and microbiological features of yeast isolates recovered from the oral cavity of 150 Iranian patients with hematological malignancies. **Design:** MALDI-TOF MS, 21-plex PCR, and rDNA sequencing were used for identification. Antifungal susceptibility testing (broth microdilution, CLSI M27-A3/S4) and genotypic diversity of yeast isolates (amplified fragment length polymorphism) were assessed. **Results: **Nystatin treatment resulted in 70% therapeutic failure and administration of 150 mg fluconazole (FLZ) + nystatin for patients with OC relapse showed 70% clinical failure. Previous history of OC was significantly correlated with FLZ treatment requirement and nystatin failure (*P* = 0.005, α < 0.05). *Candida albicans* (80.3%) and *Kluyveromyces marxianus* (*C. kefyr*) (12.7%) were the two most prevalent yeast species isolated. FLZ and AMB exhibited the highest geometric mean values. 21-PCR showed 98.9% agreement with MALDI-TOF MS. *K. marxianus* isolates had the same genotype, while *C. albicans *isolates grouped in 15 genotypes. **Conclusions:** Marked rate of therapeutic failure of nystatin necessitated OC treatment with systemic antifungals. *K. marxianus* was the second most prevalent yeast and 21-plex PCR could be considered as an inexpensive identification tool.

In recent decades, we have witnessed an increase in the number of patients prone to develop candidiasis [1,2]. According to epidemiological studies, more than 100 million people develop mucosal-surface fungal infections annually [3] and among these candidiasis is the most prevalent [4]. High-risk patients suffering from oral candidiasis (OC) not only experience a lower life quality [5], but importantly they may develop life-threatening invasive candidiasis, where the causative agents enter the bloodstream and cause candidemia [6,7]. It is well-known that various immunocompromised patient populations, such as those infected with HIV [8], with inherited genetic disorders, and cancer patients undergoing chemotherapy are among the most susceptible individuals to develop OC [5,9–11]. Indeed, meta-analysis studies proved that chemotherapy and radiotherapy treatments are independent risk factors for the development of OC [5,9,10].

Previous studies indicated that *C. albicans* is still the most common agent of OC, whereas non-*albicans Candida* (NAC) species, although with a lower frequency, are increasingly encountered in clinical settings [12,13]. Unfortunately, the majority of these species either intrinsically are less responsive to azole drugs [14] or they acquire resistance to antifungal agents [15]. As a result, accurate identification at the species level is of paramount importance to initiate a timely and appropriate therapeutic regimen. Nowadays, Sanger sequencing and matrix-assisted laser-desorption time-of-flight mass spectrometry (MALDI-TOF MS), owing to comprehensivity and accuracy, are being widely used in clinical settings of developed countries (European countries) [16]. However, high costs required for the purchase and maintenance of these devices hamper their use in developing countries [17,18]. In contrast, not only commercial PCR devices cost significantly less ($2 K–$4 K), but also the development of affordable and reproducible PCR devices with initial costs of $130 contributed to the popularity and extensive use of such a device in developing countries [19]. Therefore, PCR has been recommended as a reliable identification platform by the World Health Organization (WHO) [20]. Currently, Arastehfar et al., developed a comprehensive multiplex PCR that identifies the most prevalent yeast species causing infections in human [21].

Despite extensive studies on various immunocompromised populations [22–24], patients with hematological disorders were less studied in Iran. Moreover, none of the previous studies from Iran, extensively and systematically investigated the clinical outcomes that might lead to raising awareness among of clinicians. As a result, the purpose of this study was to investigate the clinical outcome, distribution, antifungal susceptibility, and genotypic diversity of yeast species isolated from the oral cavity of patients suffering from hematological disorders with oral candidiasis hospitalized in a referral hospital (Firoozgar, Tehran, Iran). Moreover, due to the lack of MALDI-TOF MS equipment in Iran, an alternative comprehensive multiplex PCR assay, 21-plex PCR, was evaluated for its identification accuracy.

## Material and methods

### Study design

One-hundred and 50 patients suffering from hematological malignancies undergoing chemotherapeutic regimens (1 December 2017 to 31 May 2018) retrospectively were enrolled in this study. All patients were admitted in the teaching hospital of Firoozgar, Tehran, Iran, which is affiliated with the Iran University of Medical Sciences (Tehran, Iran). Adults (>18 years old) without limitations in sex and the hematologic malignancy types (acute lymphocytic leukemia, chronic lymphocytic leukemia, acute myeloid leukemia, and chronic myeloid leukemia) were recruited to this study.

### Patients’ characteristics

All patients were examined by specialist clinicians for signs and symptoms of OC. Patients that met the following criteria were considered as OC positive, (a) presence of thrush (pseudomembranous) in the oral cavity, and (b) obtaining positive yeast growth from swab samples [25]. Oral mucositis was defined by observing erythematous and ulcerative lesions and all stages of mucositis (mild, moderate and severe) were included [26]. This study was reviewed by ethical committee members of the Iran University of Medical Sciences (IUMS) and ethical approval was granted (IR.IUMS.FMD.REC.1397.098). Informed consent was obtained from all patients whose identity was anonymized to researchers through the use of a numerical code identifier (1–150). Various demographic information, such as age, gender, type of cancer, sampling season (to observe the possible seasonality pattern) [27], clinical symptoms of OC, and previous history of OC were recorded for OC positive patients.

### Treatment options

In the neutropenic phase (absolute neutrophil count <500 cells/mcl), patients were prophylactically treated with Ciprofloxacin (500 mg BD/orally and Acyclovir (400 mg/TDS/orally)) [28]. All patients initially underwent treatment with topical nystatin and those with relapse were treated with the combination therapy of 150 mg fluconazole (FLZ) and topical nystatin for one week, which is not recommended by National Comprehensive Cancer Network (NCCN) clinical practice guideline [28]. Off note, owing to the research nature of this study, authors did not have any authority for the choice of antifungal agents used to treat patients recruited to this study. The respective decisions were made solely by in-charge clinicians.

### Clinical specimens processing and culture conditions

Symptomatic patients were examined and positive OC cases were confirmed by specialist resident clinicians. Sterile cotton swabs, samples were taken from the tongue and buccal mucosal lesions of patients and immediately transferred into falcon tubes containing sterile phosphate buffer saline (PBS 1×).

Initially, samples were subjected to direct microscopic examination to observe the yeast structures, followed by streaking on Sabouraud dextrose agar medium (SDA, Sigma–Aldrich, USA) and incubated at 35°C for 48 h. As some oral samples might harbor more than a single species, and in order to obtain pure colonies for each species, they were streaked on CHROMagar (CHROMagar *Candida*, France) and incubated for 48 h at 35°C. As results obtained from the aforementioned conventional assays (direct microscopy and CHROM agar) were not definitive, hence, their respective phenotypic were not further analyzed and the identity of each isolate was determined by LSU rDNA sequencing, MALDI-TOF MS, and 21-plex PCR.

### Identification of *candida* species and DNA extraction

Yeast isolates were identified by Bruker MALDI-TOF MS (MicroFlex-LT, Bremen, Germany) using a previously described full extraction method [29]. Scores > 2 indicated successful identification to the species level, and scores < 2 were regarded as correct identification at the genus level. Prior to proceeding with PCR identification, DNA samples were extracted by a CTAB method [30]. Using yeast species obtained from this study, the efficacy of a previously described 21-plex PCR [21] was evaluated. Species with failed identification in the first multiplex PCR were further tested by the second multiplex PCR and in case of obtaining negative results, isolates were checked by a third multiplex PCR [21]. PCR products were run on 2% agarose gel, stained with GelRed (BioTium, USA) and visualized with a gel documentation device (Gel Doc XR^+^, BioRad, USA). As the gold standard technique, emerging *Candida* isolates were further checked by sequencing of the D1/D2 domain of a large subunit of the rDNA gene using LROR and LR5 primers [31]. Mixed samples identified as *C. albicans* and *C. dubliniensis* by 21-plex were further tested using previously described hyphal-wall protein 1 primers [32].

### Genotyping by AFLP

In order to assess the genotypic diversity of all isolates, they were subjected to a previously published AFLP protocol [33]. AFLP data were analyzed by Bionumerics software V7.6 (Applied Math Inc., Austin, Texas).

### Antifungal susceptibility testing

The broth microdilution method of the Clinical and Laboratory Standards Institute (CLSI, M27-A3/S4) was used for antifungal susceptibility testing [34,35]. Eight routinely used antifungal drugs, including amphotericin B (AMB) (Sigma Chemical Corporation, St. Louis, MO), fluconazole (FLZ) (Pfizer, New York), itraconazole (ITZ) (Santa Cruz Biotech, Dallas), voriconazole (VOR) (Pfizer, New York), posaconazole (PSZ) (MSD, Kenilworth, USA), 5-fluorocytosine (5-FC) (Sigma Aldrich, Steinheim, Germany), caspofungin (CAS) (Merck & Co., Inc.), and anidulafungin (AND) (Pfizer, New York), were used. Plates were incubated at 35°C for 24 h and visual data were recorded. *C. krusei* ATCC 6258 and *C. parapsilosis* ATCC 22019 were used for quality control purposes. Depending on species, clinical breakpoints and epidemiological cut-off values were determined [35]. For isolates of *C. albicans*, the MIC values of FLZ, ITZ, VRZ, CAS, and AND were interpreted based on clinical breakpoints (CBP), while PSZ, AMB and 5-FC MIC values were evaluated based on epidemiological cut-off values (ECV) [35]. As for *C. tropicalis*, the MIC values of FLZ, VRZ, AND, and CAS were evaluated based on CBP and the rest of the antifungals (PSZ, ITZ, AMB, and 5-FC) based on ECV [35]. The MIC values of all antifungal agents obtained for the isolates of *K. marxianus* and *C. dubliniensis*, were evaluated based on ECV [35]. MIC_50_ for all antifungals were defined as the minimum concentration of drugs to inhibit 50% of fungal growth, whereas for amphotericin B the MIC endpoint was considered as the lowest concentration that inhibited 100% of fungal growth compared to the drug-free control.

### Statistical analyses

All the data presented in this study were analyzed by SPSS software v.24 (Windows, Chicago, IL). Statistical analysis included Pearson correlation Chi-square T-test, Eta correlation coefficient (for nominal and continuous variables), and Fei and Kramarz (for two nominal variables).

## Results

### Patients and isolates

The present study included 150 patients with hematological disorders, among them 85 patients among them 85 patients developed OC. The majority of OC patients were men (*n*= 53; 62.4%). Women represented almost one-third of the patients (*n*= 32; 37.6%). The median age of the OC patients was 53 years. Acute myeloid leukemia (AML) with the prevalence of 53% constituted the most dominant hematological malignancy followed by chronic lymphocytic leukemia (CLL) (*n*= 17; 20%), acute lymphocytic leukemia (ALL) (*n*= 15; 18%), and chronic myeloid leukemia (CML) (*n*= 7; 8%). The vast majority of the patients (*n*= 51; 60%) were prescribed with chemotherapeutic combination therapy of arsenic trioxide and cytarabine and the rest of the patients with ritoximab/cyclophosphamide (*n*= 15; 17.7%), cyclophosphamide (*n*= 13; 15.3), and fluorouracil (*n*= 6; 7.1). Erythema, inflammation and burning sensation of the buccal mucosa and tongue were the most common complaints that were detected in 59 patients (69.4%). *C. albicans* (*n*= 69; 80.3%) was the most prevalent causative agent of OC, followed by *K. marxianus* (= *Candida kefyr*) (*n*= 11; 12.7%), *C. tropicalis* (*n*= 3; 3.5%), *C. dubliniensis* (*n*= 2; 2.3%), and *Hanseniaspora opuntiae* (*n*= 1; 1.2%). One of the patients concurrently was colonized with both *C. albicans* and *H. opuntiae*.

### Lack of efficacy of nystatin for clearance of OC

Primarily all patients underwent treatment with topical nystatin, which resulted in OC clearance in approximately 30% (*n*= 28; 32.9%) of the cases. Subsequently, the rest of the patients (*n*= 57; 67.1%) were treated with a combination of 150 mg of FLZ and topical nystatin, among them 63% (*n*= 36) experienced at least one episode of OC relapse. A significant correlation was noticed between the previous experience of OC and treatment with FLZ (α < 0.05, P = 0.005) and therapeutic failure with nystatin (P < 0.05).

### Identification

**Figure 1.**
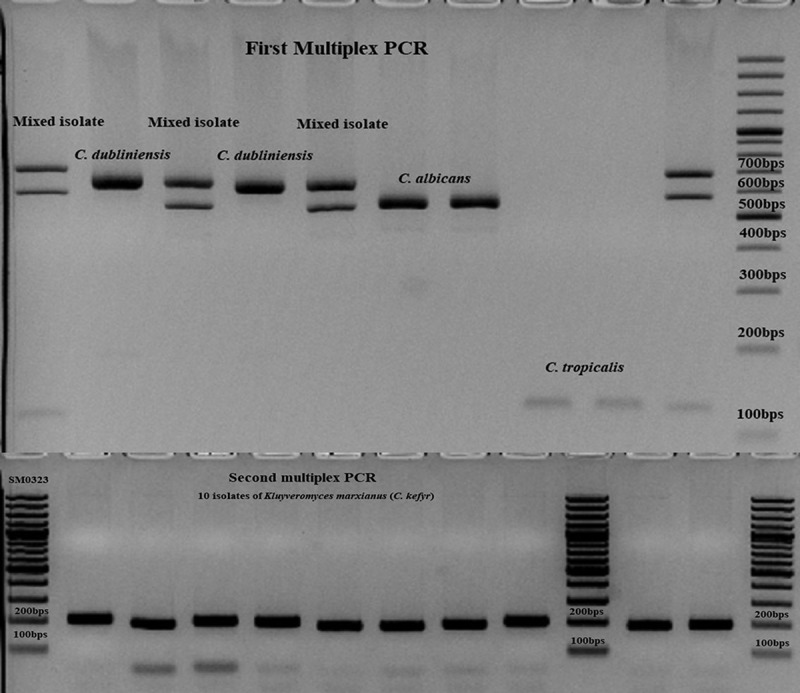
Application of 21-plex PCR for identification of clinical isolates. The majority of the strains were identified in the first multiplex PCR (*C. albicans, C. dubliniensis*, and *C. tropicalis*), the isolates of *K. marxianus* (*C. kefyr*) by the second multiplex PCR, and the only isolate of *Hanseniaspora opuntiae* was not identified by any of the PCR reactions.

MALDI-TOF MS consistent with LSU rDNA sequencing successfully identified all isolates to the species level (green score; >2), while the 21-plex PCR could not identify the only isolate of *H. opuntiae*. Interestingly, in agreement with primers targeting hyphal wall protein-1 [32], two isolates showed double bands representing both *C. albicans* and *C. dubliniensis*, while both MALDI-TOF MS and sequencing identified both as *C. albicans* (Figure 1). Those mixed samples on CHROMagar yielded the same color as *C. albicans* (green-colored colonies). As for the mixed sample containing both isolates of *C. albicans* and *H. opuntiae*, green and cream-colored colonies were observed on CHROMagar, respectively.

### AFLP genotyping

Although, infections due to *C. albicans* are mainly endogenous, some studies have shown cases of hospital-acquired infections for this species [36]. Accordingly, the high prevalence of *C. albicans* and *K. marxianus* in the oral cavities of patients included in this study, convinced us, to perform the AFLP for all isolates (except for *H. opuntiae, n*= 1) to observe the genotypic diversity. Moreover, all of the patients enrolled in this study were from the same hospital and the same unit (blood unit). One of the most important findings of this study was encountering a high number of *K. marxianus* isolates (*n*= 11; 12.7%) and through AFLP those isolates belonged to the same genotype (Supplementary Figure 1). As for the isolates of *C. albicans*, we found three major clusters (C), including C1 (*n*= 16), C2 (*n*= 17) and C3 (*n*= 22) and 12 minor and unique genotypes each containing a single isolate. No significant relationships were found between genotype, the season of isolation, and hospitalized ward. Two DNA samples of *C. albicans* isolates did not yield any banding patterns (probably due to the presence of inhibitors), therefore they were excluded from the final AFLP figure.

### Antifungal susceptibility testing

Antifungal susceptibility results and MIC values are represented in Table 1. Among isolates of *C. albicans*, only 2.8% (*n*= 2) were resistant to FLZ (≥ 8 µg/ml) and 4 isolates (5.7%) were intermediate for ITZ (0.25–0.5 µg/ml), while they were all either susceptible to VRZ (≤ 0.12 µg/ml), CAS (≤ 0.25 µg/ml), and AND (≤ 0.25 µg/ml) or wild-type (WT) for AMB (≤ 2 µg/ml), 5-FC (≤ 0.5 µg/ml), and PSZ (≤ 0.06 µg/ml). All of the *K. marxianus* isolates showed WT phenotype for all antifungals used [5-FC (≤ 0.5 µg/ml), AMB (≤ 2 µg/ml), FLZ (≤ 1 µg/ml), ITZ (≤ 0.12 µg/ml), PSZ (≤ 0.25 µg/ml), VRZ (≤ 0.015 µg/ml), AND (≤ 0.25 µg/ml), and CAS (≤ 0.0.3 µg/ml)]. All isolates of *C. tropicalis*, except for two isolates that were intermediate for ITZ (0.25–0.5 µg/ml), were susceptible to FLZ (≤ 2 µg/ml), VRZ (≤ 0.12 µg/ml), AND (≤ 0.25 µg/ml), and CAS (≤ 0.25 µg/ml) and WT for PSZ (≤ 0.12 µg/ml), AMB (≤ 2 µg/ml), and 5-FC (≤ 0.5 µg/ml). WT phenotype and low MIC values against all antifungal agents were noticed for the two *C. dubliniensis *isolates  and the only isolate of *H. opuntiae*, respectively. Two isolates of *C. dubliniensis* were WT. For all of the tested antifungal agents these isolates and the only isolate of *H. opuntiae* exhibited low MIC values.CAS, VRZ, and AND, and showed the lowest geometric mean values for both *C. albicans* and *K. marxianus*, while FLZ and AMB showed the highest GM mean values for *C. albicans* and *K. marxianus* isolates, respectively.

**Table 1.** Antifungal susceptibility testing against yeast species isolated from oral lesions of patients with hematological malignancies.

		ECV^a^ (µG/ml)		CBP^b^ (µG/ml)					
Organism	Antifungal	<ECV	>ECV	S	SDD	I	R	GM	Range
*C. albicans* (*n*= 69)	AMB	69	0	NA	NA	NA	NA	0.439	0.12–1
	FLZ	67	2	67	0	NA	2	0.461	0.125–8
	ITZ	65	4	65	4	NA	0	0.046	0.016–0.5
	VRZ	53	16	69	NA	0	0	0.022	≤0.015–0.06
	PSZ	69	0	NA	NA	NA	NA	0.026	≤0.015–0.06
	CAS	69	0	100%	NA	0	0	0.019	≤0.008–0.12
	AND	68	1	100%	NA	0	0	0.026	0.015–0.12
	5-FC	69	0	NA	NA	NA	NA	0.060	<0.06–0.06
*K. marxianus* (*n*= 11)	AMB	10	0	NA	NA	NA	NA	1.149	0.012–2**^C^**
	FLZ	10	0	NA	NA	NA	NA	0.326	0.12–0.5
	ITZ	10	0	NA	NA	NA	NA	0.053	0.06–0.25**^d^**
	VRZ	9	1	NA	NA	NA	NA	0.016	0.015–0.03
	PSZ	10	0	NA	NA	NA	NA	0.028	0.015–0.03
	CAS	10	0	NA	NA	NA	NA	0.014	≤0.008–0.03
	AND	10	0	NA	NA	NA	NA	0.024	0.015–0.12
	5-FC	10	0	NA	NA	NA	NA	0.056	<0.06–0.12**^e^**
*C. tropicalis* (*n*= 3)	AMB	3	0	NA	NA	NA	NA	NA	0.12–0.5
	FLZ	3	0	3	0	NA	0	NA	0.25–1
	ITZ	3	0	NA	NA	NA	NA	NA	0.03–0.25
	VRZ	1	2	1	NA	2	0	NA	≤0.008–0.25
	PSZ	3	0	NA	NA	NA	NA	NA	≤0.015–0.06
	CAS	3	0	3	NA	0	0	NA	≤0.008–0.06
	AND	3	0	3	NA	0	0	NA	0.015–0.06
	5-FC	3	0	NA	NA	NA	NA	NA	≤0.12–0.25
*C. dubliniensis* (*n*= 2)	AMB	2	0	NA	NA	NA	NA	NA	0.03–0.25
	FLZ	2	0	NA	NA	NA	NA	NA	0.06–0.25
	ITZ	2	0	NA	NA	NA	NA	NA	0.015–0.03
	VRZ	2	0	NA	NA	NA	NA	NA	<0.008–0.03
	PSZ	2	0	NA	NA	NA	NA	NA	0.06–0.25
	CAS	2	0	NA	NA	NA	NA	NA	0.008–0.06
	AND	2	0	NA	NA	NA	NA	NA	0.015–0.06
	5-FC	2	0	NA	NA	NA	NA	NA	0.06–0.12
*H. opuntiae* (*n*= 1)	AMB (0.5)	NA	NA	NA	NA	NA	NA	NA	0.5
	FLZ (1)	NA	NA	NA	NA	NA	NA	NA	1
	ITZ (0.03)	NA	NA	NA	NA	NA	NA	NA	0.03
	VRZ (0.015)	NA	NA	NA	NA	NA	NA	NA	0.015
	PSZ (0.015)	NA	NA	NA	NA	NA	NA	NA	0.015
	CAS (0.03)	NA	NA	NA	NA	NA	NA	NA	0.03
	AND (0.015)	NA	NA	NA	NA	NA	NA	NA	0.015
	5-FC (0.06)	NA	NA	NA	NA	NA	NA	NA	0.06

a. Epidemiological cut-off value b. Clinical breakpoint c. For the isolates of *K. marxianus*, the epidemiological cut-off value of >2 were considered resistant to AMB d and e. For the isolates of *K. marxianus*, the epidemiological cut-off value of ≥0.5 were considered resistant to ITZ and 5-FC S; Susceptible, SDD; Susceptible dose-dependent, I; Intermediate, R; Resistant, NA; Not applicable Geometric mean value was not calculated for species containing less than 10 isolates. The only isolate of *H. opuntiae* showed low MIC values for all antifungal agents, which have are mentioned next to each antifungal used.

## Discussion

The worrisome escalation of cancer incidence may facilitate the emergence of OC in this population of patients. Unfortunately, despite a high annual burden of OC and the possible link with the development of candidemia [10,37] and even cancer [38], many clinical and microbiological aspects of this complication remained to be elucidated. Conducting local, national, and worldwide studies might result in a better clinical outcome, ease of management of OC cases, establishing appropriate empiric therapy, and finally lower hospital costs. Consequently, in order to bridge the gap between the lack of knowledge on clinical outcome and microbiological features of OC among patients suffering from hematological disorders we conducted this study in Iran.

In our study, more than half (56%) of the enrolled patients were positive for OC, from whom 86 yeast isolates were recovered. Encountering such a high number of OC positive patients is likely due to the fact that all our patients underwent chemotherapy [5], which is known to be the main risk factor for the manifestation of OC [5]. *C. albicans* (81%) was the most encountered *Candida* species, followed by *K. marxianus* (12%), *C. tropicalis* (3.5%), *C. dubliniensis* (2.3%) and *H. opuntiae* (1.2%). It was not surprising that *C. albicans* was the most prevalent *Candida* species, as this fungus is the most abundant yeast agent inhabiting mucosal surfaces of the gastrointestinal (GI), pulmonary, and urinary tract [27,39]. Predominance of *C. albicans* is in agreement with other studies [40–42], while unlike those studies we found *K. marxianus* as the second most frequently encountered yeast species. Previous studies have shown that patients with hematological disorders are prone to develop candidiasis due to *K. marxianus* [27,43], which corroborates the high rate of this yeast species in our study. Together with Jamel et al., we reported the second clinical case of *H. opuntiae* [44]. Studies of English abstracts published from 2009 to 2018 revealed an increasing trend in the spectrum and/or prevalence of emerging *Candida* and yeast species isolated from the oral cavity of Iranian HIV positive and cancer patients [22,45–48]. The noted differences in the distribution of diverse yeast species found in various studies might reflect the variations in patient subgroups, diet, sample types, or even the administered antifungal regimen. In line with those facts, application of informative and useful next-generation sequencing platforms have revealed that smoking [49], hygiene, and even the composition of the tap water [50] may cause microbial diversity in the oral microbiome.

Approximately 30% of our patients were successfully treated with nystatin and the remaining required combination of 150 mg of FLZ + topical nystatin, which indicates insufficient efficiency of this drug for clearance of OC. Interestingly, we observed a significant correlation between previous occurrence of OC and the requirement of more than one period of FLZ + nystatin treatment and nystatin therapeutic failure. Some studies showed a superiority of nystatin pastilles or pastille and suspension over nystatin alone, whilst the adverse side effects on the gastrointestinal tract, unpleasant taste, use of high doses and extended time of prescriptions are the drawbacks of nystatin formulations [51]. On the contrary, other studies have shown a similar efficacy of nystatin to that of placebo and an inferiority to FLZ when used in severe immunodeficient patients [52]. Although, we did not test nystatin in our antifungal susceptibility panel, other studies have shown high MIC values for topical agents [48] and recommended the use of systemic antifungal drugs, such as FLZ [5,53]. Moreover, randomized, double-blind clinical trials have shown a higher efficacy of FLZ and MCF when they were used in HIV-positive patients suffering from OC [8,54]. In agreement with other studies that showed a superiority of a single dose of 750 mg of FLZ rather than two-week-long 150 mg FLZ therapy in HIV-positive individuals [8], we found that almost 70% of patients treated with 150 mg FLZ and topical nystatin therapy showed OC relapse on more than one occasion. Adopting such a single high dose of FLZ regimen instead of a standard two-week long FLZ therapy of 150 mg, might result in lower risk of development of FLZ-resistant isolates stemming from a more extensive exposure time [55]. In case of FLZ-refractory cases, utilization of other azole agents, including PSZ, VRZ, ITZ [5], and micafungin [19] have been recommended [5]. This is in line with our findings that ITZ, VRZ, and PSZ showed the lowest geometric mean values and FLZ the highest. Nowadays, due to the expanding spectrum of yeast species in clinical settings that are possibly less responsive to azoles, it might be useful to implement such a therapeutic regimen of systemic antifungals for patients suffering from OC.

Approximately, 3% of the isolates of *C. albicans* were resistant to FLZ and infected patients underwent treatment with FLZ + nystatin, which is in concordance with other studies that previous exposure with FLZ results in the emergence of FLZ resistant isolates in patients suffering from candidemia [56]. Although a newer generation of antifungals targeting CYP51A have shown promising results when tested in animal models infected with *C. auris* [57], *Cryptococcus neoformans* [44], *Cocciodiodes posadasii* and *Coccidioides immitis* [58], their efficacy remains to be evaluated for the treatment of OC patients. Moreover, if proved to be efficient when tested in human OC positive cases, utilization of these drugs in clinical settings might contribute in preserving the limited systemic antifungal armamentarium and reducing the burden of systemic drug-resistant yeast species.

With regards to the identification of our clinical yeast isolates, MALDI-TOF MS and 21-plex showed 100% and 99% consistency with LSU rDNA sequencing, respectively. Although rare, MALDI-TOF MS successfully identified *H. opuntiae*, while the 21-plex PCR did not. Previous studies have shown the superiority of MALDI-TOF MS for identification of clinically important yeast species [59,60], but unfortunately, this device is not available in Iran as a developing country. However, PCR as an affordable and reproducible device increasingly proves to be a popular identification platform in developing countries [19,20]. Moreover, samples with mixed species could be differentiated via 21-plex PCR, while neither MALDI-TOF MS nor Sanger sequencing were able to differentiate the respective yeast species.

Subjecting our isolates to AFLP revealed that all 11 isolates of *K. marxianus* were in the same cluster. That may suggest that these isolates originated from the same source. Unfortunately, as a limitation of our study, we could not find the source of the *K. marxianus* isolates. However, the lack of dairy products in the hospital diet of our patients, precludes the fact that they have acquired *K. marxianus* during their hospitalization stay. Moreover, it is even likely that outside the hospitalization period those patients might have consumed dairy products containing *K. marxianus* and subsequently were infected with this yeast.

Unexpectedly, we found 15 genotypes of *C. albicans*, including three major clones constituting approximately 82% of *C. albicans* isolates and 12 minor genotypes each represented by a single isolate. As the three major genotypes encompassed more than 80% of the *C. albicans* isolates, the phenomenon of intrahospital transmission might be likely. Despite some studies using AFLP have ruled out the intrahospital transmission phenomenon for *C. albicans* isolates recovered from blood samples [61], others using pulse-field gel electrophoresis (PFGE) proved otherwise [36]. Encountering with such a contradictory finding might stem from the variation in the technology used, but it is noteworthy that both AFLP and PFGE possess a high discriminatory power. Unfortunately, similar to the isolates of *K. marxianus*, we did not carry out any screening protocols to prove the concept of intrahospital transmission for the *C. albicans* isolates, which is the main limitation of our study. Although, AFLP proved its superiority and higher resolution over multi-locus sequence typing (MLST) [61], application of platforms with a higher resolution, such as next-generation sequencing, might provide a better explanation for the intrahospital transmission phenomenon of *C. albicans* isolates.

## Conclusion

Clinical outcome data derived from our study was compelling enough to persuade the clinicians to abandon the usage of nystatin as a choice of antifungal therapy for the treatment of OC positive patients with hematological malignancies. Interestingly, *K. marxianus* after *C. albicans* represented the second most prevalent yeast species isolated from the oral cavity of our patients, and 21-plex proved its efficiency for identification of clinically encountered yeast species. Moreover, through performing AFLP, we found that the majority of *C. albicans* and 100% of *K. marxianus* isolates showed the same genotypic background that might be an indication of intrahospital transmission. However, proving this fact requires environmental screening to find the same clinical source, which is one of the shortages of our study.
