# Supplementary figures and images for: Incidence and spectrum of yeast species isolated from the oral cavity of Iranian patients suffering from hematological malignancies
Source: J Oral Microbiol. 2019 Apr 12;11(1):1601061. doi: 10.1080/20002297.2019.1601061 (PMC6484487; doi:10.1080/20002297.2019.1601061)

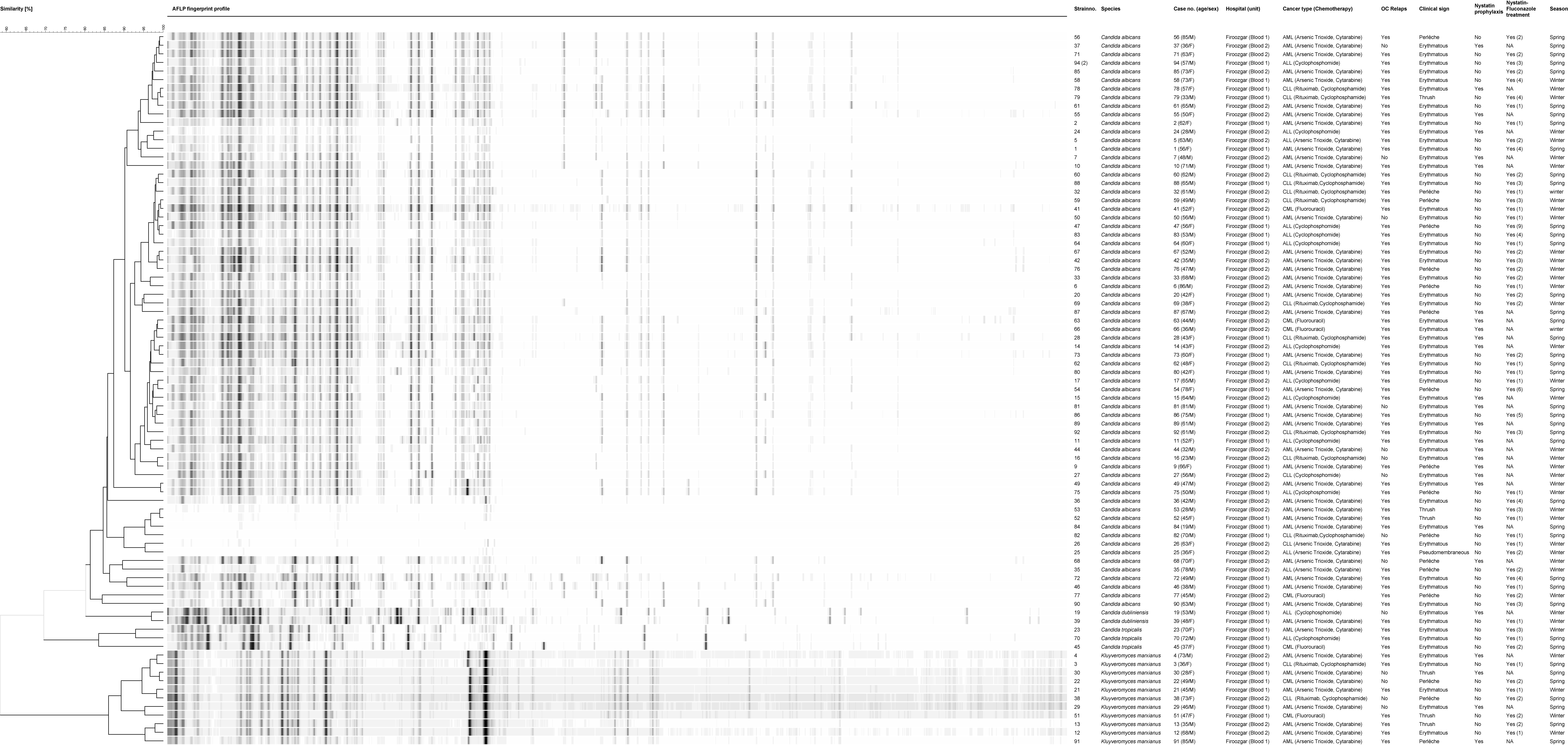

Supplement: Supplemental Material [file ZJOM_A_1601061_SM4652.tif]
